# Advances in Visualization Tools for Phylogenomic and Phylodynamic Studies of Viral Diseases

**DOI:** 10.3389/fpubh.2019.00208

**Published:** 2019-08-02

**Authors:** Kristof Theys, Philippe Lemey, Anne-Mieke Vandamme, Guy Baele

**Affiliations:** Department of Microbiology, Immunology and Transplantation, Rega Institute for Medical Research, Clinical and Epidemiological Virology, KU Leuven, Leuven, Belgium

**Keywords:** visualization, phylogenetics, phylogenomics, phylodynamics, infectious disease, epidemiology, evolution

## Abstract

Genomic and epidemiological monitoring have become an integral part of our response to emerging and ongoing epidemics of viral infectious diseases. Advances in high-throughput sequencing, including portable genomic sequencing at reduced costs and turnaround time, are paralleled by continuing developments in methodology to infer evolutionary histories (dynamics/patterns) and to identify factors driving viral spread in space and time. The traditionally static nature of visualizing phylogenetic trees that represent these evolutionary relationships/processes has also evolved, albeit perhaps at a slower rate. Advanced visualization tools with increased resolution assist in drawing conclusions from phylogenetic estimates and may even have potential to better inform public health and treatment decisions, but the design (and choice of what analyses are shown) is hindered by the complexity of information embedded within current phylogenetic models and the integration of available meta-data. In this review, we discuss visualization challenges for the interpretation and exploration of reconstructed histories of viral epidemics that arose from increasing volumes of sequence data and the wealth of additional data layers that can be integrated. We focus on solutions that address joint temporal and spatial visualization but also consider what the future may bring in terms of visualization and how this may become of value for the coming era of real-time digital pathogen surveillance, where actionable results and adequate intervention strategies need to be obtained within days.

## 1. Virus Epidemiology and Evolution

Despite major advances in drug and vaccine design in recent decades, viral infectious diseases continue to pose serious threats to public health, both as globally well-established epidemics of e.g., Human Immunodeficiency Virus Type 1 (HIV-1), Dengue virus (DENV) or Hepatitis C virus (HCV), and as emerging or re-emerging epidemics of e.g., Zika virus (ZIKV), Middle East Respiratory Syndrome Coronavirus (MERS-CoV), Measles virus (MV), or Ebola virus (EBOV). Efforts to reconstruct the dynamics of viral epidemics have gained considerable attention as they may support the design of optimal disease control and treatment strategies (1, 2). These analyses are able to provide answers to questions on the diverse processes underlying disease epidemiology, including the (zoonotic) origin and timing of virus outbreaks, drivers of spatial spread, characteristics of transmission clusters and factors contributing to enhanced viral pathogenicity and adaptation (3–5).

Molecular epidemiological techniques have proven to be important and effective in informing public health and therapeutic decisions in the context of viral pathogens (6, 7), given that most of the viruses with a severe global disease burden are characterized by high rates of evolutionary change. These genetic changes are being accumulated in viral genomes on a time scale similar to the one where the dynamics of population genetic and epidemiological processes can be observed, which has lead to the definition of viral phylodynamics as the study of how epidemiological, immunological, and evolutionary processes act and potentially interact to shape viral phylogenies (8). As such, phylogenetic trees constitute a crucial instrument in studies of virus evolution and molecular epidemiology, elucidating evolutionary relationships between sampled virus variants based on the temporal resolution in the genetic data of these fast-evolving viruses that allows resolving their epidemiology in terms of months or years. Through the integration of population genetics theory, epidemiological data and mathematical modeling, insights into epidemiological, immunological, and evolutionary processes shaping genetic variation can be inferred from these phylogenies. The field of phylodynamics has generated new opportunities to obtain a more detailed understanding of evolutionary histories—through time as well as geographic space—and transmission dynamics of both well-established viral epidemics and emerging outbreaks (9, 10).

The ability of molecular epidemiological analyses, and phylodynamic analyses in particular, to fully exploit the information embedded in viral sequence data has significantly improved through a combination of technological innovations and advances in inference frameworks during the past decades. From a data perspective, genomic epidemiology is becoming a standard framework driven by high-throughput sequencing technologies that are associated with reduced costs and increasing turnover. Moreover, the portability and potential of rapid deployment on-site of these new technologies enable the generation of complete genome data from samples within hours of taking the samples (11). This rising availability of whole-genome sequences increases the resolution by which historical events and epidemic dynamics can be reconstructed. From a methodological perspective, new developments in statistical and computational methods along with advances in hardware infrastructure have allowed the analysis of ever-growing data sets, the incorporation of more complex models and the inclusion of information related to sample collection, infected host characteristics and clinical or experimental status (generally known as metadata) (9, 10, 12, 13).

In contrast to a marked increase in the number of software packages targetting increasingly efficient but complex approaches to infer annotated phylogenies by exploiting genomic data and the associated metadata, the intuitive and interactive visualization of their outcomes has not received the same degree of attention, despite being a key aspect in the interpretation and dissemination of the rich information that is inferred. Phylogenies are typically visualized in a rather simplistic manner, with the concept of depicting evolutionary relationships using a tree structure already illustrated in Charles Darwin's notebook (1837) and his seminal book “The Origin of Species” (14). Early phylogenetic tree visualization efforts constituted an integral part of phylogenetic inference software packages and as such were restricted to simply showing the inferred phylogenies on a command line or in a simple text file, often even without an accompanying graphical user interface. The longstanding use of phylogenies in molecular epidemiological analyses has however led to the emergence of increasingly feature-rich visualization tools over time. The advent of the new research disciplines such as phylogenomics and phylodynamics necessitated more complex visualizations in order to accommodate projections of pathogen dispersal onto a geographic map, ancestral reconstruction of various types of trait data and appealing animations of the reconstructed evolution and spread over time. Tree visualizations resulting from these analyses are also complemented by visual reconstructions of other important aspects of the model reconstructions, such as population size dynamics over time, transmission networks and estimates of ancestral states for traits of interest throughout the tree (15).

Across disciplines, adequate visualizations are pivotal to communicate, disseminate and translate research findings into meaningful information and actionable insights for clinical, research and public health officials. The aim to improve data-driven decision making fits within a broader scope to establish a universal data visualization literacy (16). To this end, enhancing collaborations and dissemination of visualizations is increasingly achieved through sharing of online resources for hosting annotated tree reconstructions (17), online workspaces (18) and continuously updated pipelines that accommodate increasing data flow during infectious disease outbreaks (19) (see further sections for more information and examples of these packages). Given the plethora of options for presenting and visualizing results, and its importance for effectively communicating with a wide audience, choosing the appropriate representation and visualization strategy can be challenging. Recent work on this topic focuses on navigating through all the available visualization options by offering clear guidelines on how to turn large datasets into compelling and aesthetically appealing figures (20).

## 2. A Framework for Visualization

A large array of software packages for performing phylogenetic and phylodynamic analyses have emerged in the last decade, in particularly for fast-evolving RNA viruses [see (10) for a recent overview]. A more recent but similar trend can be seen for methodologies and applications aimed at visualization of the output of these frameworks. In addition to the need to communicate these outputs in a visual manner, an increasing recognition for the added value of adequate visualization for surveillance, prevention, control and treatment of viral infectious diseases has resulted into the merging of data analytics and visualization, with the visualization aspect being increasingly considered as an elementary component within all-round analysis platforms. This review illustrates the evolution in phylogenetically-informed visualization modalities for evolutionary inference and epidemic modeling based on viral sequence data, evolving from an initial purpose to serve basic interpretation of the results to an in-depth translation of complex information into usable data for virologists, researchers and public health officials alike. Novel features and innovative approaches often stem from a community need, which can be translated into a specific challenge to be addressed by current and future software applications. Throughout this article, we discuss some of the major bottlenecks for interpretation and visualization of phylodynamic results, and subsequently solutions that have addressed or can address these challenges.

A closer inspection of how tools for manipulation, visualization and interpretation of evolutionary scenarios have steadily grown over time reveals different trends of interest. First, visualization needs for phylodynamic analyses are very heterogeneous in nature, driven by the intrinsic objective to better understand viral disease epidemiology. Due to the increasing complexity and interactivity of the various aspects that make up phylodynamic analyses, the gradual change in visualization tools has resulted in a wide but incomplete range of solutions provided (illustrated by the Wikipedia list of phylogenetic tree visualization software^1^). Software applications for phylodynamic analyses have extended into investigations of population dynamics over time, trait evolution and spatio-temporal dispersal, while still using a phylogenetic tree as their core concept. While we will focus predominantly on the concept of a phylogenetic tree as the backbone of phylodynamic visualization, these analyses also produce other types of output that go beyond visualizing phylogenies, especially when it comes to trait data reconstruction. Second, the continuing advances in visualization—which try to keep up with increasing complexities in the statistical models employed—not only result in more features being available for end users to exploit, they may also come at an increased cost in terms of usability and responsiveness. Formats for input and output files have increased in complexity, from simple text files to XML specifications and (Geo)JSON file formats for geographical features. Reading, understanding and editing such files may prove to be a challenging task for practitioners. However, most visualization tools do not expose these complexities to their users and offer an intuitive point-and-click interface and/or drag-and-drop functionality for customizing the visualization (18). Despite such intuitive interactivity, intricate knowledge and a certain amount of programming/scripting experience is often required for those users who want to customize and/or extend their visualization beyond what the application has to offer. Third, visualization goals tend to become context-dependent in that not all phylodynamic analyses deal with the same sense of urgency, with established epidemics requiring different prevention and treatment strategies than outbreak detection and surveillance. For example, in established epidemics (e.g., HIV-1) thefocus may be on identifying (important) clusters within a very large phylogeny (17), whereas analyses in ongoing outbreaks often determine whether newly generated sequences correspond to strains of the virus known to circulate in a certain region and try to establish spillover from animal reservoirs (21). Finally, despite the major achievements so far, visualization tools are reaching the limits of their capacity to comprehensibly present analysis results of large datasets. Promising developments and strategies are becoming available that move visualization beyond the goal of communicating and synthesizing results, and actively play an important role in providing analytics to better understand evolutionary and demographic processes fueling viral dispersal and pathogenicity.

## 3. Visualization Challenges and Solutions

Phylogenetic tree visualizations have played a central role since the earliest evolutionary and molecular epidemiological analyses of fast-evolving viral pathogens. The first computer programs aimed at constructing phylogenies [e.g., PAUP^*^; (22, 23), and PHYLIP; (24)] were only equipped with minimal tree drawing and printing facilities, limited by the available operating systems and programming languages of that time. Standalone, phylogenetically-oriented programs [e.g., MUST; (25) and later on Treeview; (26)] were specifically developed to interact with tree reconstruction output and to ease tree editing and viewing. Even as phylogenetic inference became inherently more sophisticated, for example with the development of Bayesian phylogenetic inference and the release of initial versions of MrBayes (27) which contained sophisticated search strategies to ensure finding the optimal set of phylogenetic trees, these software packages still contained their own text-based tree visualization component(s).

However, over time a wide range tree visualization software has been released, offering a continuous increase of tree visualization and manipulation functionalities. These packages have been developed as either standalone software packages or have been integrated into larger data management and analysis platforms [e.g., MEGA (28)]. The numerous all-round programs available to date offer a range of similar basic tree editing capabilities including the coloring and formatting of tree nodes, edges and labels, the addition of numerical or textual annotations, searching for specific taxa as well as the re-rooting, rotation and collapsing of clades. Different tree formats can be imported and again exported to various textual and graphical formats (e.g., vector-based formats: portable document format (pdf), encapsulated postscript (eps), scalable vector graphics (svg), …). A limited set of applications provide more advanced visualization functionalities that enable interactive visualization and management of highly customized and annotated phylogenetic trees. Nevertheless, major hurdles still exist that hinder adequate communication and interpretation of phylodynamic analyses. These hurdles mainly relate to the scalability of the visualization, highlighting uncertainty associated with the results and the interactive rendering of available metadata. Recent innovative developments attempt to tackle these bottlenecks, although some tools are specifically directed toward addressing a single (visualization) challenge. We here provide an overview of such challenges, along with examples of figures generated by software packages that aim to tackle these challenges. Note that all of our visualization examples are shown in the *Evolving visualization examples* section below.

First, a major challenge is the ever-increasing size of data sets being analyzed, leading to difficulties with navigating through the resulting phylogenetic trees and to problems with interpreting the inferred dynamics, not only from a computational perspective (e.g., to render large images in a timely manner) but also from the human capability to deal with high levels of detail. Software packages that mainly aim to visualize phylogenetic trees as well as those that target more broad analyses have implemented various solutions to accommodate systematic exploration of large phylogenies. Dendroscope (29) was one of the first visualization tools aimed at large phylogenies, with its own format to save and reopen trees that had been edited graphically, offering a magnifier functionality to focus on specific parts of the (large) phylogeny. Follow-up versions (30) focused on rooted phylogenetic trees and networks, and offered parallel implementations of demanding algorithms for computing consensus trees and consensus networks to increase responsiveness. Phylo.io (31) improves the legibility of large trees by automatically collapsing nodes so that an overview of the tree remains visible at any given time. iTOL [(18), but see below] and IcyTree (32) also provide intuitive panning and zooming utilities that make exploring large phylogenetic trees of many thousands of taxa feasible. The PhyloGeoTool [(17); also see **Figure 4**] eases navigation of large trees by performing an *a priori* iterative clustering of subtrees according to a predefined diversity ratio, as well as pre-rendering the visualization of those subtrees enabling fluent navigation. PastML (33) allows visualizing the tree annotated with reconstructed ancestral states as a zoomable HTML map based on the Cytoscape framework (34). PastView (35) offers synthetic views such as transition maps, integrates comparative analysis methods to highlight agreements or discrepancies between methods of ancestral annotations inference, and is also available as a webserver instance. Grapetree (36) initially collapses branches if there are more than 20,000 nodes in the tree and then uses a static layout that splits the tree layout task into a series of sequential node layout tasks. With the development of many packages targetting the visualization of large phylogenies in recent years, the question arises whether they will continue to be maintained and extended with novel features.

**Figure 1:**
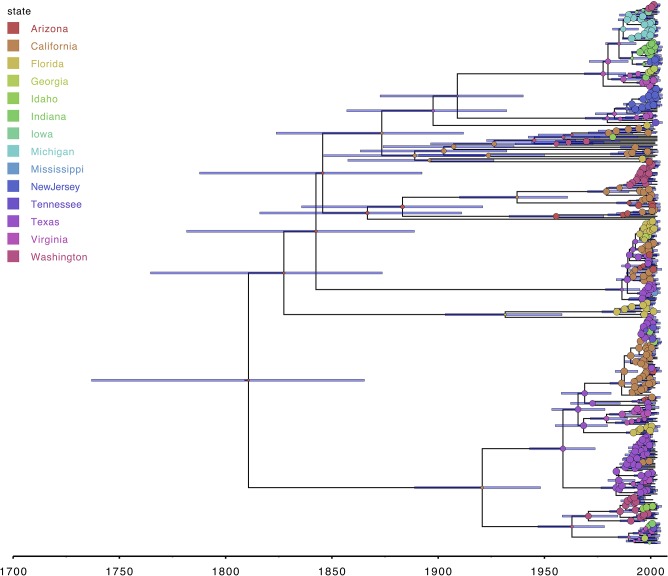
FigTree allows visualizing various tree formats, including maximum clade credibility trees from Bayesian phylogenetic analyses (38). External and internal nodes can easily be annotated using the information in the source tree file, and the time information within the tree allows adding a time axis which facilitates interpretation. Annotations shown here for the RABV data set are the 95% highest posterior density (HPD) age intervals and the most probable ancestral location state at each internal node, with the circle width corresponding to the posterior support for the internal location state reconstruction.

A second challenge lies with the fact that phylogenies represent hypotheses that encompass different sources of error, and the extent of uncertainty at different levels should be presented accordingly. Bootstrapping (37) and other procedures are often used to investigate the robustness of clustering in estimated tree topologies,. Numerical values that express the support of a cluster are generally shown on the internal nodes of a single consensus summary tree [e.g., FigTree; (38)] or by a customized symbol [e.g., iTOL; (18)]. Although conceptually different, posterior probabilities on a maximum clade credibility (MCC) tree, majority consensus tree or other condensed trees from the posterior set of trees resulting from Bayesian phylogenetic inference can be shown in a similar manner. An informative and qualitative approach to represent the complete distribution of rooted tree topologies is provided by DensiTree [(39); also see **Figure 10**], which draws all trees in a set simultaneously and transparently, and the different output visualizations highlight various aspects of tree uncertainty. For time-scaled phylogenetic trees, uncertainty in divergence time estimates of ancestral nodes (e.g., 95% highest posterior density (HPD) intervals) is usually displayed with a horizontal (node) bar (see **Figure 1** for an example). Additionally, ancestral reconstructions of discrete or continuous trait states at the inner nodes of a tree are increasingly facilitated by various probabilistic frameworks, and these inferences are also accompanied by posterior distributions describing uncertainty. To visualize this uncertainty, PastML (33) inserts pie charts at inner nodes to show likely states when reconstructing discrete traits such as the evolutionary history of drug resistance mutations, while SpreaD3 (40) is able to depict uncertainty of continuous traits, e.g., as polygon contours for (geographical) states at the inner nodes [see (40) for an example]. Much like the visualization packages that focus on large phylogenies (see above), the applications listed here have their own specific focus with sometimes limited overlap in functionality.

A third challenge consists of the visual integration of metadata with phylogenetic trees—often in the form of either a discrete and/or continuous trait associated with each sequence—which is in part related to the previous challenge concerning uncertainty of trait reconstructions. Incorporating virus trait information (e.g., drug resistance mutations, treatment activity scores) or host characteristics (e.g., gender, age, risk group) in phylogenetic inference can substantially facilitate the interpretation for end users and accelerate the identification of potential transmission patterns. Tree reconstruction and visualization software generally share a set of basic operations for coloring taxa, branches or clades according to partial or exact label matches. While these annotations can be performed manually using a graphical user interface, this can be time-consuming and is prone to errors. Hence, several software programs offer functionalities to automate the selection and annotation of clades of interest, for example through the use of JavaScript libraries [e.g., PhyD3; (41), SpreaD3; (40)]—also see **Figure 3**—or Python toolkits [e.g., ETE; (42), Baltic; (43)]. Alternatively, drag-and-drop functionality of plain text annotation files generated with user-friendly text editors facilitate this process, as is for example the case in iTOL (18). These scripting visualization frameworks also foster more intense tree editing through their functionalities to annotate inner nodes, clades and individual leaves with charts (pie, line, bar, heatmap, boxplot), popup information, images, colored strips and even multiple sequence alignments. Even more advanced integration efforts entail the superimposition of tree topology with layers of information on geographical maps, such as terrain elevation, type of landcover and human population density [e.g., R package seraphim; (44, 45)].

Finally, visualization and accompanying interpretation are a critical component of infectious disease epidemiological and evolutionary analyses. Indeed, many researchers use visualization software during analyses for data exploration, identifying inconsistencies, and refining their data set to ensure well-supported conclusions regarding an ongoing outbreak. As such, the visualizations themselves are gradually refined and improved over the course of a research project, with the final figures accompanying a publication often being post-processed versions of the default output of a visualization package or customly designed to attract a wide audience, both through the journal's website and especially social media [see e.g., (5)]. On the other hand, the advent of one-stop platforms [MicroReact; (46) and Nextstrain; (19, 47), also see **Figure 5**] that seamlessly connect the different steps of increasingly complex analyses and visualization of genomic epidemiology and phylodynamics allows automating this process. Applications that are exclusively tailored toward tree manipulation and viewing are starting to offer management services and registration of user accounts [iTOL; (18)], while command-line tools (Gotree; https://github.com/evolbioinfo/gotree) aimed at manipulating phylogenetic trees and inference methods (PASTML; (33) increasingly enable exporting trees that can directly be uploaded to iTOL, supporting the automation of scripting and analysis pipelines.

## 4. Evolving Visualization Examples

**Figure 2:**
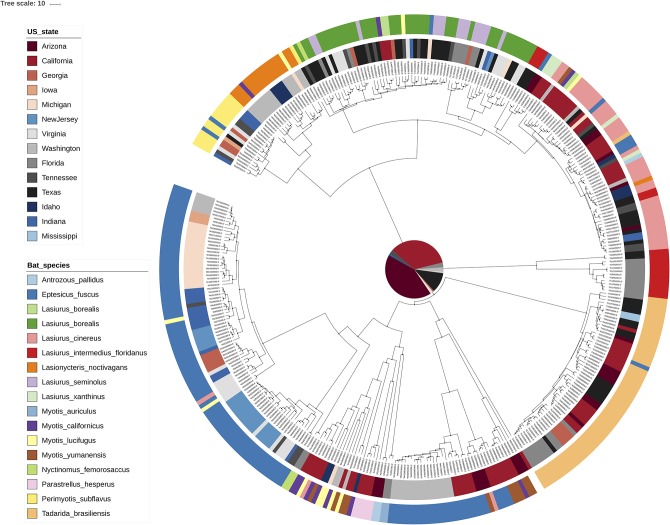
Interactive tree of life [iTOL; (18)] visualization of the MCC tree for RABV. Rather than exploiting the annotations within an MCC tree, iTOL allows importing external text files with annotations through an easy drag-and-drop interface. We have here colored the tip nodes according to the bat host species (outer circle) as well as the sampling location (inner circle) corresponding to each sample. Many visual aspects can be set this way and an extensive online help page is available.

**Figure 3:**
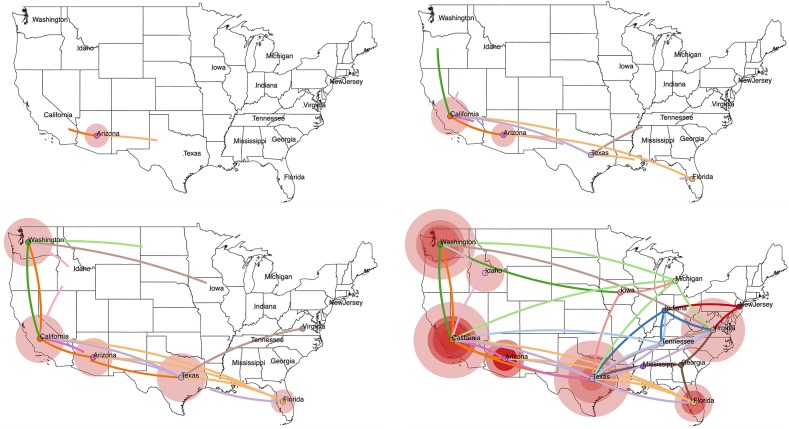
Projecting an MCC tree onto a geographic map using SpreaD3 (40). In a discrete phylogeography setting, as is the case here, the ancestral location states are combined with coordinates corresponding to the states in the US from which the RABV samples were obtained. We use centroid coordinates for the US states to enable this visualization. SpreaD3 animates the reconstructed virus dispersal over time, and we here show four snapshots (starting from the estimated origin of the epidemic at the root node) that capture the reconstructed dispersal over time and geographic space, i.e., in 1860, 1940, 1980, and the “present” (mid 2005). The transitions between the US states are colored according to the US state of destination for that particular transition, whereas the size of the circles around a location is proportional to the number of lineages that maintain that location.

**Figure 4:**
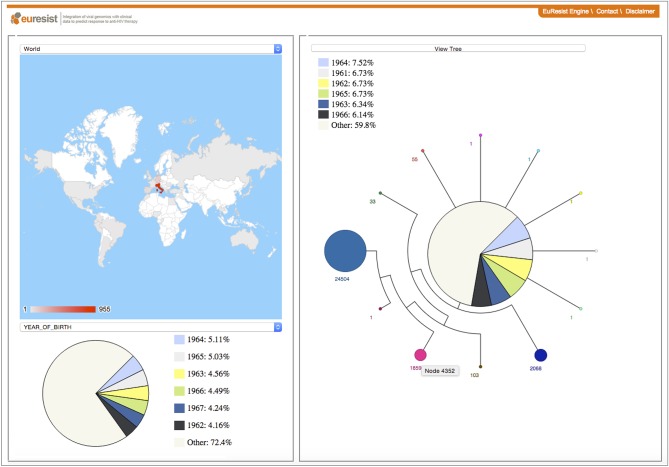
The PhyloGeoTool offers a visual approach to explore large phylogenetic trees and to depict characteristics of strains and clades—including for example the geographic context and distribution of sampling dates—in an interactive way (17). A progressive zooming approach is used to ensure an efficient and interactive visual navigation of the entire phylogenetic tree.

**Figure 5:**
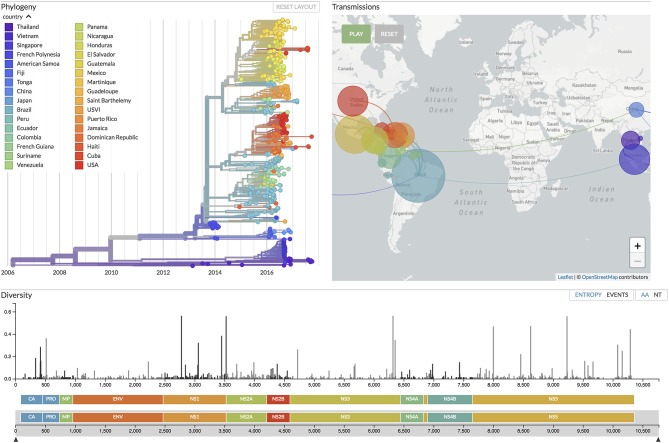
Focusing on real-time tracking of several viruses, Nextstrain (19) provides up-to-date visualizations of phylogenetic and phylogeographic analyses, the latter with an animation over time similar to SpreaD3 (40). Shown here is the current situation for Zika virus evolution, based on analyses of 506 genomes sampled between February 2013 and September 2017 from 32 countries around the world (see the figure legend), corresponding to samples taken in 6 different regions of the world: China, Southeast Asia, Japan and Korea, Oceania, South America, and North America.

**Figure 6:**
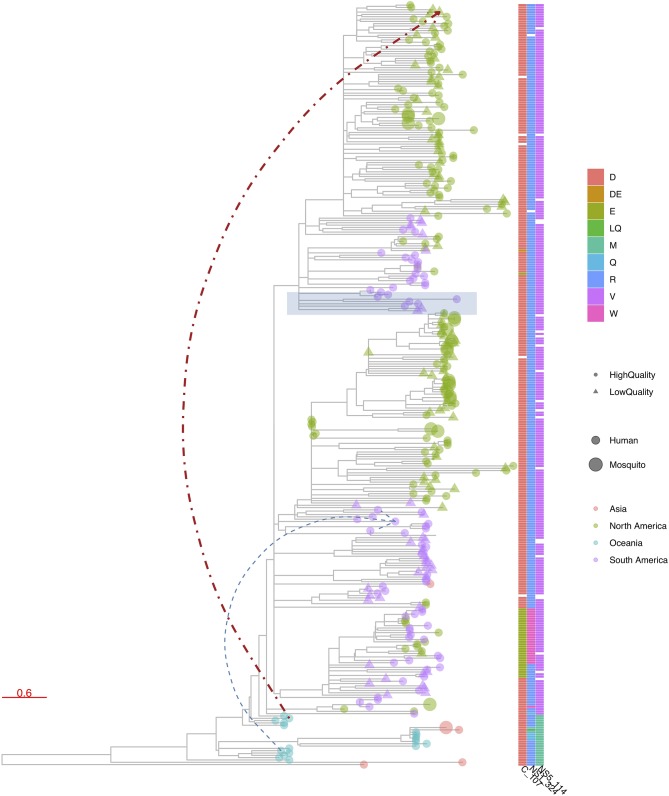
R package ggtree (48) visualization of a phylogenetic tree constructed from publicly available Zika virus (ZIKV) genomes. ggtree allows similar advanced customized visualization of phylogenetic trees as e.g., iTOL, but by means of the traditional R scripting language. In this figure, tree leaves are colored according to continent of sampling, with a size corresponding to the host status and shape indicating the completeness of the CDS, using a cutoff of 99% of nucleotide positions being informative. A heatmap was added to denote the presence of amino acid mutations at three chosen genome positions. Finally, a particular clade was highlighted in blue based on a given internal node and two additional links between chosen taxa were added.

**Figure 7:**
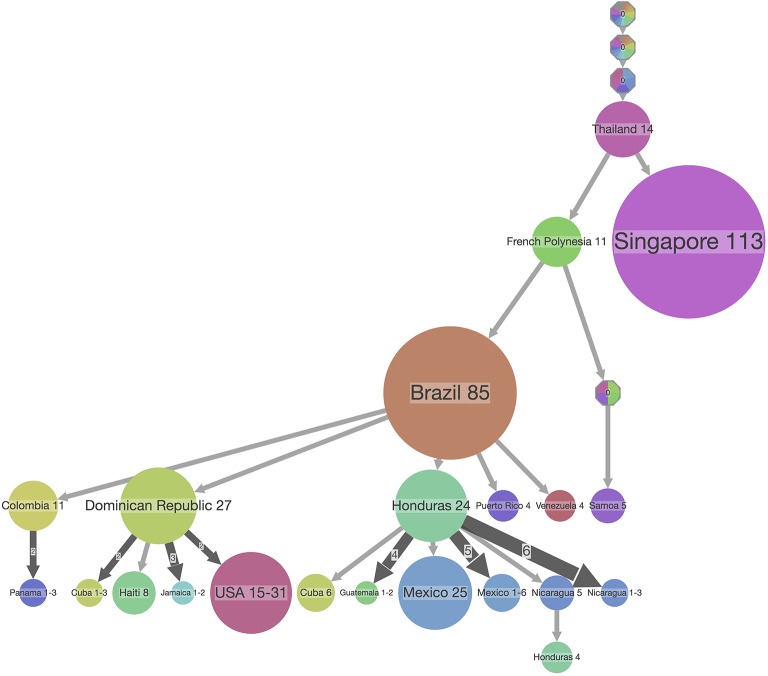
PASTML summary visualization of the ancestral reconstruction of state locations of the ZIKV dataset used in Figure 6. The top-down visualization corresponds to an iterative clustering starting from the root of the tree at the top, with the size of the dot corresponding to the number of taxa in a clade which share the same ancestral state which is indicated on top of the dot. In this type of visualization, a compressed representation of the ancestral scenarios is visualized that highlights the main facts and hides minor details by performing both a vertical and horizontal merge [but see (33)]. The branch width corresponds to the number of times its subtree is found in the initial tree, and the circle size at a tip is proportional to the size of the compressed (or merged) cluster.

**Figure 8:**
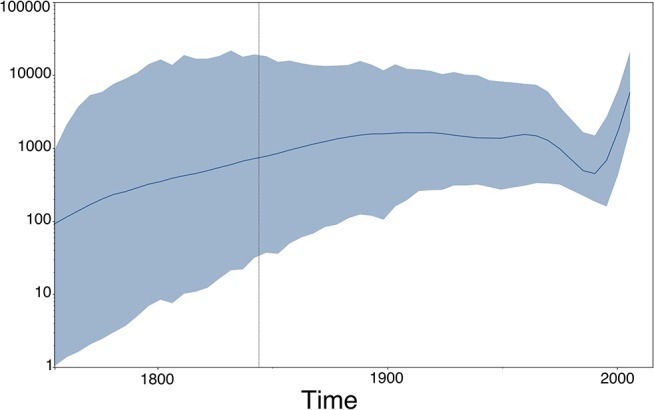
Population (size) dynamics over time visualization of our RABV analysis (previous section) using Tracer (49). This type of output does not directly depend upon the estimated MCC tree, but rather on the estimated (log) population sizes of the Skygrid model (50), which are provided in a separate output file by BEAST (51).

**Figure 9:**
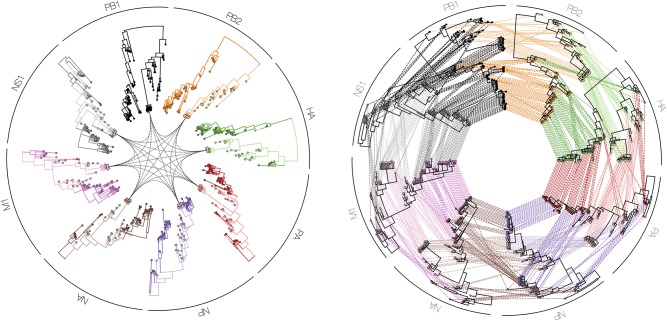
Tanglegrams are typically shown in a side-by-side manner, in order to easily and visually identify differences in clustering between two or more phylogenetic trees, for example when inferred from different influenza proteins (PB1, PB2, PA, HA, NP, NA, M1, and NS1). Such a series of trees can also be visualized in a circle facing inwards with a particular isolate highlighted in all plotted phylogenies **(left figure)**, or with all isolates interconnected between all proteins **(right figure)**.

**Figure 10:**
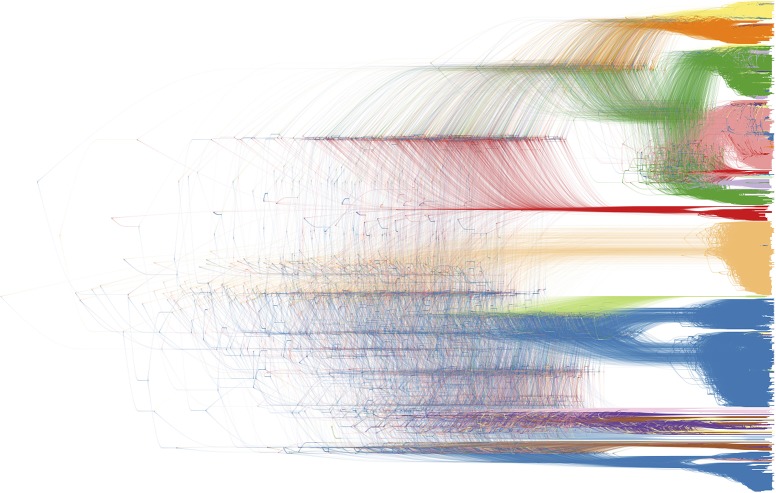
Bayesian phylogenetic inference software packages generate a large number of posterior trees, potentially annotated with inferred ancestral traits. This collection of trees is often summarized using a consensus tree, allowing to plot a single tree with posterior support values on the internal nodes. DensiTree enables drawing all posterior trees in the collection; areas where a lot of the trees agree in topology and branch lengths show up as highly colored areas, while areas with little agreement show up as webs (39). We refer to Figure 2 for the color legend of the host species, as the legend drawn by DensiTree was not very readable and could not be edited (in terms of its textual information).

In the previous sections, we have already covered a wide range of software packages for visualizing phylogenetic trees as well as their associated metadata, which may or may not be used in a joint estimation of sequence and trait data [for an overview of integrating these data types in various inference frameworks for pathogen phylodynamics, we refer to (9)]. We here organize our visualization examples into different broader categories: different approaches toward visualizing associated trait data with a focus on phylogeography (Figures 1–3), browser-based online applications (Figures 4, 5), applications that use existing libraries such as those available in R, Python and JavaScript for example (Figures 6, 7), non-phylogenetic visualizations typically associated with pathogen phylodynamics (Figure 8), and finally custom-written code or applications that focus on assessing phylogenetic uncertainty (Figures 9, 10).

As a first example, we illustrate the development of innovative visualization software packages on the output of a Bayesian phylodynamic analysis of a rabies virus (RABV) data set consisting of time-stamped genetic data along with two discrete trait characteristics per sequence, i.e., the sampling location—in this case the state within the United States from which the sample originated—and the bat host type. This RABV data set comprises 372 nucleoprotein gene sequences from North American bat populations, with a total of 17 bat species sampled between 1997 and 2006 across 14 states in the United States (52). We used BEAST 1.10 (51) in combination with BEAGLE 3 (13) to estimate the time-scaled phylogenetic tree relating the sequences, along with inferring the ancestral locations of the virus using a Bayesian discrete phylogeographic approach (53) and, at the same time, infer the history of host jumping using the same model approach. Upon completion of the analysis, we constructed a maximum clade credibility (MCC) tree from the posterior tree distribution using TreeAnnotator, a software tool that is part of the BEAST distribution. This MCC tree contains at its internal nodes the age estimates of all of the internal nodes, along with discrete probability distributions for the inferred location and host traits at those internal nodes.

Figure 1 shows the visualization of the MCC tree in FigTree, with internal nodes annotated according to the posterior ancestral location state probabilities within the MCC tree file. As expected, one can observe that posterior support for the preferred ancestral location decreases from the observed tips toward the root, in other words the further we go back in time, the more uncertain the inferred location states become. All of the information required to make the FigTree visualization in Figure 1 is contained within a NEXUS file, containing all of the ancestral trait annotations, which we use as the (only) input for the FigTree (38). The standard Newick file format itself does not contain such trait annotations but remains in popular use for storing phylogenetic trees and is hence supported by most (if not all) phylogenetic visualization packages. In general however, Newick and other older formats (e.g., NEXUS) offer limited expressiveness for storing and visualizing annotated phylogenetic trees and associated data, which has lead to extensions for this format being proposed [e.g., the extended Newick format; (54)]. FigTree allows users to upload annotation information for the sequences in the analyzed alignment in the form of a simple tab-delimited text file, and a parsimony approach can be used to infer the most parsimonious state reconstruction for the internal nodes from those provided for the tips. iTOL (18) is another application that can take an MCC tree as its input file and allows annotating branches and nodes of the phylogenetic tree using descriptions provided through the use of simple text files in which custom visualization options can easily be declared (Figure 2). iTOL is even suited for showing very large trees (with more than 10,000 leaves) with Webkit-based browsers—such as Chromium/Google Chrome, Opera and Safari—offering the best performance.

Newer input/output file formats for phylogenetic trees and their accompanying annotations, including the XML-based standards PhyloXML (55) and NeXML (56), have the benefit of being more robust for complex analyses and easier to process and extend. In particular, applications of phylodynamics aimed at reconstruction and interpretation of spatio-temporal histories have become broadly and increasingly applied in viral disease investigations. The incorporation of geographical and phylogenetic uncertainty into molecular epidemiology dynamics is now well-established (53, 57), and dedicated developments from a visualization perspective have soon followed to accommodate the outcomes of these models. Early attempts include the mapping of geo-referenced phylogenetic taxa to their geographical coordinates [e.g., GenGis; (58), Cartographer; (59)], while more recent efforts of joint ancestral reconstruction of geographical and evolutionary histories enable visual summaries of spatial-temporal diffusion through the interactive cartographic projection using GIS- and KML-based virtual globe software (60). The latest developments generalize toward interactive web-based visualization of any phylogenetic trait history and are based on data-driven documents (D3) JavaScript libraries and the JSON format to store geographic and other tree-related information (40). As an example, we have created a web-based visualization of our analyzed RABV data set by loading the obtained MCC tree into the SpreaD3 (40) phylodynamic visualization software package (see Figure 3). SpreaD3 actually consists of a parsing and a rendering module, with the former obtaining the relevant information out of the MCC tree and the latter converting this information into a (Geo)JSON file format, potentially in combination with a geographic map, which can easily be downloaded from websites offering GeoJSON files of different regions of the world and with different levels of detail. The generated output consists of an in-browser animation that allows tracking a reconstructed epidemic over time using a simple slider bar, with the possibility to zoom into specific areas of the map. In Figure 3, we show the reconstructed spread of RABV across the United States at four different time points throughout the epidemic, starting with the estimated location of origin in the state of Arizona and tracking the RABV spread as it disperses to all of the 14 states in our data set.

The SpreaD3 visualization in Figure 3 is an example of an increasing trend toward web-based software tools that can run in any modern browser, making them compatible with all major operation systems, without requiring any additional software packages to be installed by the user. A distinction can be made between browser-based tools that are able to work without internet access [Phylocanvas; (http://phylocanvas.org), phylotree.js; (61), IcyTree; (32), SpreaD3; (40), PhyloGeoTool; (17), see Figure 4] or that are only accessible online [iTOL; (18), phylo.io; (31)]. Web-based visualization platforms enhance collaborations and output dissemination in a very efficient and simple manner through their ability to share web links of complex and pre-annotated tree visualizations. Transferring genomic data and associated data to an online service may invoke privacy issues which is not the case for tools that execute data processing purely on the client side. By contrast, online accessible visualization tools such as iTOL (18) offer tree management possibilities to organize and save different projects, annotated datasets and trees for their users. These online packages typically also provide export functionalities to facilitate the production of publication-quality and high-resolution illustrations [see also MrEnt; (62), Mesquite; (59)], directed toward end-users with minimal programming experience.

SpreaD3 also illustrates the growing movement toward animated visualizations over time and (geographic) space and as such focuses entirely on the visualization aspect of pathogen phylodynamics. Recently, entire pipelines have emerged that include data curation, analysis and visualization components, with Nextstrain as its most popular example (19). On the data side, Python scripts maintain a database of available sequences and related metadata, sourced from public repositories as well as GitHub repositories and other (more custom-made) sources of genomic data. Fast heuristic tools enable performing phylodynamic analysis including subsampling, aligning and phylogenetic inference, dating of ancestral nodes and discrete trait geographic reconstruction, capturing the most likely transmission events. The accompanying Nextstrain website (https://nextstrain.org/) provides a continually-updated view of publicly available data alongside visualization for a number of pathogens such as West Nile virus, Ebola virus, and Zika virus. For the latter virus, we provide the currently available data visualization in Nextstrain (at time of submission) in Figure 5, showing a color-coded time-scaled maximum-likelihood tree alongside an animation of Zika geographic transmissions over time as well as the genetic diversity across the genome. Analysis of such outbreaks relies on public sharing of data, and Nextstrain has taken the lead to address data sharing concerns by preventing access to the raw genome sequences, and by clearly indicating the source of each sequence, while allowing derived data—such as the inferred phylogenetic trees—to be made publicly available. We note that these animated visualizations by their very nature do not easily yield publication-ready figures, requiring alternative approaches to be devised. Animations resulting from software packages such as SPREAD, SpreaD3 and Nextstrain can be hosted on the authors' website or they can be captured into a video file format and uploaded as supplementary materials onto the journal website. Alternatively, screenshots of the animation can be taken at relevant time points throughout the visualization and subsequently post-processed to include in the main or supplementary publication text.

Finally, browser-based packages such as SpreaD3 employ JavaScript libraries (e.g., D3) to produce dynamic, interactive data visualizations in web browsers, known specifically for allowing great control over the final visualization. Custom programs are also typically written in R as a long list of popular R libraries are readily available, with ggplot2 quickly rising to popularity and finding use in both R and Python languages. A system for declaratively creating graphics based on *The Grammar of Graphics* (63), ggplot2 was built for making professional looking figures with minimal programming efforts. Figure 6 shows an example of ggtree, which extends ggplot2 and is designed for visualization and annotation of phylogenetic trees with their covariates and other associated data (48). A recent software package that is implemented in JavaScript and Python is PastML (33), which uses the Cytoscape.js library (64) for visualizing phylogenetic trees (Figure 7). Given that these types of libraries contain many tried-and-tested functions that save substantial time when creating novel software packages, future visualization efforts are likely to see increased usage of readily available visualization libraries in programming languages such as R, Python and JavaScript.

## 5. Other Common Visualizations in Phylodynamics

Phylogenies reconstructed from viral sequence data and their corresponding annotated tree-like drawings and animations lie at the heart of many evolutionary and epidemiological studies that involve phylogenomics and phylodynamics applications. Additional graphical output can be generated using visualization packages that focus on other aspects than the estimated phylogeny, but that are however in some manner dependent on the phylogeny. Coalescent-based phylodynamic models that connect population genetics theory to genomic data can infer the demographic history of viral populations (65), and plots of the effective population sizes over time—such as the one shown in Figure 8 for our RABV data set, which uses the Skygrid model (50) and its accompanying visualization in Tracer (49)—are commonly used to visualize the inferred past population size dynamics (50, 66, 67).

A variety of other summary statistics computed over the course of a phylogeny also benefit from visual representations, such as for the basic reproduction number and its rate of change as a function through time (68). Closely related are lineage-through-time plots (69) that allow exploring graphically the demographic signal in virus sequence data and revealing temporal changes of epidemic spread. Neher et al. (70) plotted cumulative antigenic changes over time by integrating viral phenotypic information into phylogenetic trees of influenza viruses, thereby providing additional insights into the rate of antigenic evolution compared to representations of neutralization titers that are traditionally transformed into a lower-dimensional space (71, 72). Another example relates to reconstructions of phylogeographic diffusion in discrete space, where patterns of migration links are typically projected into a cartographic context, but quantitive measures are additionally computed including the expected number of effective location state transitions (known as “Markov jumps”). Information on migrations in and out of a location state can be obtained by visualizations of the number of actual jumps between locations as well as the waiting times for each location, either as a total or proportionally over time (73–76).

The inference of transmission trees and networks (“who infected whom and when”) using temporal, epidemiological and genetic information is an application of phylodynamics that has made substantial methodological progress in the last decade (77–79). Different from phylogenetic trees that represent evolutionary relationships between sampled viruses, transmission trees describe transmission events between hosts and require visualizations that are tailored to the analysis objectives (80–82). Consensus transmission trees, such as maximum parent credibility (MPC) trees (80) or Edmonds' trees (83), visually alert the user on putative infectors (indicated with arrows), corresponding infection times and potential super-spreaders. (80) use the Cytoscape framework (34) for drawing the transmission trees, and a similar adaptation of the original biological network-oriented framework has been done by PastML (33) (see above).

Finally, in order to compare two or more trees that are estimated from the same set of virus samples, but differ in the method used for tree construction or in the genomic region considered, tanglegrams provide insightful visualizations. The most popular use case is the comparison of two trees displayed leaf-by-leaf-wise with differences in clustering highlighted by lines connecting shared tips (84). Alternatively, tanglegrams allow mapping tree tip locations to mapped geographical locations using GenGis (58, 85). The Python toolkit Baltic (https://github.com/evogytis/baltic) provides functionalities to draw tangled chains, as shown in Figure 9, which are advanced sequential tanglegrams to compare a series of trees (43, 86). The use of phylogenetic networks, which are a generalization of phylogenetic trees, can also visualize phylogenetic incongruences, which could be due to reticulate evolutionary phenomena such as recombination (e.g., HIV-1) and hybridization (e.g., influenza virus) events (30, 32, 87). Tanglegrams and related visualization of sets of trees [e.g., DensiTree (39); see Figure 10] provide a qualitative and illustrative comparison of trees, buy this may prove to be less suited for the interpretation of extremely large trees or sets of trees. Recent quantitative approaches allow the exploration and visualization of the relationships between trees in a multi-dimensional space of tree similarities, based on different tree-to-tree distance metrics that identify a reduced tree space that maximally describe distinct patterns of observed evolution [Mesquite; (88), R package treespace; (89, 90)].

## 6. Context Dependence of Visualization Requirements

We have discussed a wide range of visualization packages for phylogenetic and phylodynamic analyses that allow improving our understanding of viral epidemiological and population dynamics. While these efforts may ultimately assist in informing public health or treatment decisions, visualization needs can differ according to the type of virus epidemics studied and questions that need to be answered. For example, the required level of visualization detail is high for (re-)emerging viral outbreaks when actionable insights should be obtained in a timely fashion in order to control further viral transmissions, with real-time tracking of viral spread and the identification of sources, transmission patterns and contributing factors being key priorities (91). As a result, software packages that aim to address these questions are typically developed with an explicit emphasis on speed through the use of heuristics, and stress the importance of connectivity and interactivity to quickly respond to the availability of new data in order to develop novel insights into an ongoing epidemic. One-stop and fully-integrated analysis platforms such as MicroReact (46) and Nextstrain (19) adhere to these needs by providing the necessary visualizations of virus epidemiology and evolution across time and space, and by implementing support for collaborative analyses and sharing of genomic data and analysis outputs. A strategy of interest in these settings is the ability for phylogenetic placement of novel sequence data (92, 93), for example when updated outbreak information suggests specific cases should be investigated but the reconstruction of a new phylogeny is not desirable, as this may prove too time consuming. To avoid such *de novo* re-analyses of data sets, software tools such as iTOL (18) and PhyloGeoTool (17) offer functionalities to visualize placements of sequence data onto an existing phylogeny. A key future challenge of these approaches is to assess and visualize the associated phylogenetic placement uncertainty, or if this information would be unavailable to at least indicate the various stages in which novel sequences were added onto the (backbone) phylogeny. While methodological developments are rigorous in their accuracy assessment—for example through simulation studies—and may even provide visual options for representing the placement uncertainty [see e.g., (92)], visualization packages themselves do not offer an automated way of assessing or conveying this information and as such may project overconfidence of the power of the phylogenetic placement method used. Additionally, other flexible visualization options based on real-time outbreak monitoring can be of great interest such as highlighting locations from which cases have been reported but for which genomic data are still lacking, to clarify the potential impact of these missing data on the currently available inference results.

Investigations of more established epidemics usually involve much larger sample sizes, are more retrospective-oriented in design and incorporate more heterogeneous information, and therefore benefit from more extended visualization frameworks. For most of these globally prevalent pathogens, clinical and phenotypic information is often available and questions relate to the population- or patient-level dynamics of viral adaptation and the identification of transmission clusters. For example, the selection of the virus strain composition of the seasonal influenza vaccine is informed by analyses and visualizations of circulating strains and their antigenic properties using the nextflu framework (47, 91). Other diverse examples include investigations of the impact of country-specific public health interventions on transmission dynamics (94, 95), the identification of distinctive socio-demographic, clinical and epidemiological features associated with regional and global epidemics (96–99) and large-scale modeling of epidemiological links among geographical locations (100–102). In these settings, relevant software packages should consider the scalability of large phylogenies and allow user-friendly exploration of heterogeneous and customized annotations. Overall, it is anticipated that future work on visualization tools, accompanying analysis and visualization software developments as described above, will result in a merging of these two epidemic perspectives, with the development of context-independent visualization software tools that can handle both scenarios equally well.

## 7. Conclusions

Viral pathogens, in particular RNA viruses, have been responsible for epidemics and recurrent outbreaks associated with high morbidity and mortality in the human population, for a duration that can span from hundreds of years [e.g., HCV (103) and DENV (104)] to decades [e.g., HIV-1 (3)]. RNA viruses are known for their potential to quickly adapt to host and treatment selective pressure, but their rapid accumulation of genomic changes also provides opportunities to study their population and transmission dynamics in high resolution. Consequently, the fields of phylogenomics and phylodynamics play a pivotal role in studies on epidemiology and transmission of viral infectious diseases, and have advanced our understanding of the dynamical processes that govern virus dispersal and evolution at both population and host levels. Compared to the tremendous achievements in the performance of evolutionary and statistical inference models and hypothesis testing frameworks, software packages and resources aimed at visualizing the output of these studies have experienced difficulties to handle the increasing complexity and sizes of the analyses, for example to display levels of uncertainty and to integrate associated demographic and clinical information. Accurate and meaningful visual representation and communication are however essential tools for the interpretation and translation of outcomes into actionable insights for the design of optimal prevention, control and treatment interventions. With a plethora of applications for phylodynamics having been introduced in the last decades, in particular tailored toward reconstructions of spatiotemporal histories—which start to become useful in public health surveillance—visualization has substantially grown as an elementary discipline for investigations of infectious disease epidemiology and evolution. An extensive array of software and tools for the manipulation, editing and annotation of output visualizations in the field of pathogen phylodynamics is available to date, characterized by varying technical specifications and functionalities that respond to heterogeneous needs from the research and public health communities.

The increasing recognition for visualization tools in support of viral outbreak surveillance and control has stimulated the advent of more complex and fully integrated frameworks and platforms, all the while focusing on user experience and ease of customisation. We anticipate that future visualization developments will take further leaps in this ongoing trend by tackling remaining challenges to display increasing amounts of dense information in a human-readable manner and introducing concepts from new disciplines such as visual analytics. In particular, high expectations are stemming from the ensemble of visualization methods that allow users to work at, and move between, focused and contextual views of a data set (105). Large scientific data sets with a temporal aspect have been the subject of multi-level focus+context approaches for their interactive visualization (106), which minimize the seam between data views by displaying the focus on a specific situation or part of the data within its context. These approaches are part of an extensive series of interface mechanisms used to separate and blend views of the data, such as overview+detail, which uses a spatial separation between focused and contextual views, and zooming, which uses a temporal separation between these views (105). Phylogenetic trees can be interactively visualized as three-dimensional stacked representations (107). The field of phylogenomics and phylodynamics visualizations will increasingly implement and adapt technologies from other disciplines, as already illustrated by tools and studies using the network-oriented Cytoscape package (33, 34, 78), or through the use of virtual reality technologies including customizable mapping frameworks and high-performance geospatial analytical toolboxes. As such, concomitant to the ongoing developments in sample collection and sequencing, the design of more complex analytical inference models and powerful hardware infrastructure will be complemented by a new era in visualization applications that will collaboratively foster visualizations that track virus epidemics and outbreaks in real-time and with high resolution.

## Search Strategy

An initial but already comprehensive list of publications was compiled from backward and forward citation searches of the various visualization software packages the authors have (co-)developed, as well as those packages that the authors have used throughout their academic career. Complementing this already extensive list, we searched PubMed and Google Scholar, which keeps track of arXiv and bioRxiv submissions and hence decreased the risk of missing potential publications. Additional supplementary searches have been performed by backward and forward citation chasing of all of the included references throughout the writing process of writing the manuscript for the initial submission on April 7th 2019. No date restrictions were applied, but only visualization packages and publications written in English were considered.

## Author Contributions

KT wrote the manuscript. PL helped with the interpretation and writing. A-MV gave the idea, helped with the interpretation and writing. GB wrote the manuscript and prepared the visualizations.

### Conflict of Interest Statement

The authors declare that the research was conducted in the absence of any commercial or financial relationships that could be construed as a potential conflict of interest.

## Footnotes

^1^https://en.wikipedia.org/wiki/List_of_phylogenetic_tree_visualization_software
